# Fractured tracheostomy tube as a foreign body in a pediatric patient: a case report and review of literature

**DOI:** 10.1093/jscr/rjaf194

**Published:** 2025-04-03

**Authors:** Sultan K Kadasah, Abdulrazaq M Alshammari, Nader S Alharbi, Ibtihal S Alshehri, Raghad Y Alasiri, Abdulaziz Al Qahtani, Ali S Al Shahrani

**Affiliations:** Department of Surgery, College of Medicine, University of Bisha, Aseer, Saudi Arabia; Department of Otorhinolaryngology Head and Neck Surgery, King Salman Hospital, Riyadh, Saudi Arabia; Department of Otorhinolaryngology Head and Neck Surgery, Shaqra University, Riyadh, Saudi Arabia; Department of Otorhinolaryngology Head and Neck Surgery, King Khalid University, Aseer, Saudi Arabia; Department of Otorhinolaryngology Head and Neck Surgery, King Khalid University, Aseer, Saudi Arabia; Department of Otorhinolaryngology, Armed Forces Hospital, Khamis Mushait, Aseer, Saudi Arabia; Department of Otorhinolaryngology, Armed Forces Hospital, Khamis Mushait, Aseer, Saudi Arabia

**Keywords:** pediatric tracheostomy, tracheostomy complications, tracheostomy tube fracture, airway management

## Abstract

Tracheostomy is a common surgery in children, usually required due to chronic respiratory insufficiency or neuromuscular illnesses. While tracheostomy considerably improves respiratory control, it also increases the risk of serious consequences such as tube dislodgement and blockage. This article describes a catastrophic event in which a dislodged tracheostomy tube (TT) occurred in a 10-year-old female patient with cerebral palsy who had been on ventilator support for 5 years. The dislodged tube slipped unnoticed into the right main bronchus, causing severe respiratory distress and sudden reduction in oxygen saturation. This life-threatening condition requires rapid diagnosis and surgical management. It also reviews the relevant literature to provide insights into best practices for managing similar cases, highlighting the importance of early identification and management of rare complications to prevent life-threatening outcomes. Key takeaways from this TT fracture case report highlight the importance of vigilance in long-term patient care. Early intervention is critical for patient safety, and healthcare providers should be aware of fracture risk in patients with prolonged TT use. Regular monitoring and maintenance are essential to prevent complications. This report emphasizes the need to investigate cleaning and sterilization methods to determine their impact on TT structural integrity.

## Introduction

Tracheostomy is a common life support airway procedure [1]. It is often observed in different specialties including medical, surgical, and critical care settings. It is a safe procedure with a mortality rate of <5% [1, 2]. However, complications can occur [1]. They categorized the complications as early or late. Early complications include bleeding, wound infection, pneumothorax, and obstruction of the tracheostomy tube (TT) [2]. Late complications include tracheostomy stenosis, granulation formation, innominate artery erosion, tracheoesophageal fistula, and fracture or dislocation of the TT [1, 2]. Fracture and migration of TT are rare complications, with a low incidence rate of <1/1000 [3]. Based on this case, it is evident that the early identification and management of such rare complications are crucial, as supported by our review of the literature.

## Case presentation

**Figure 1:**
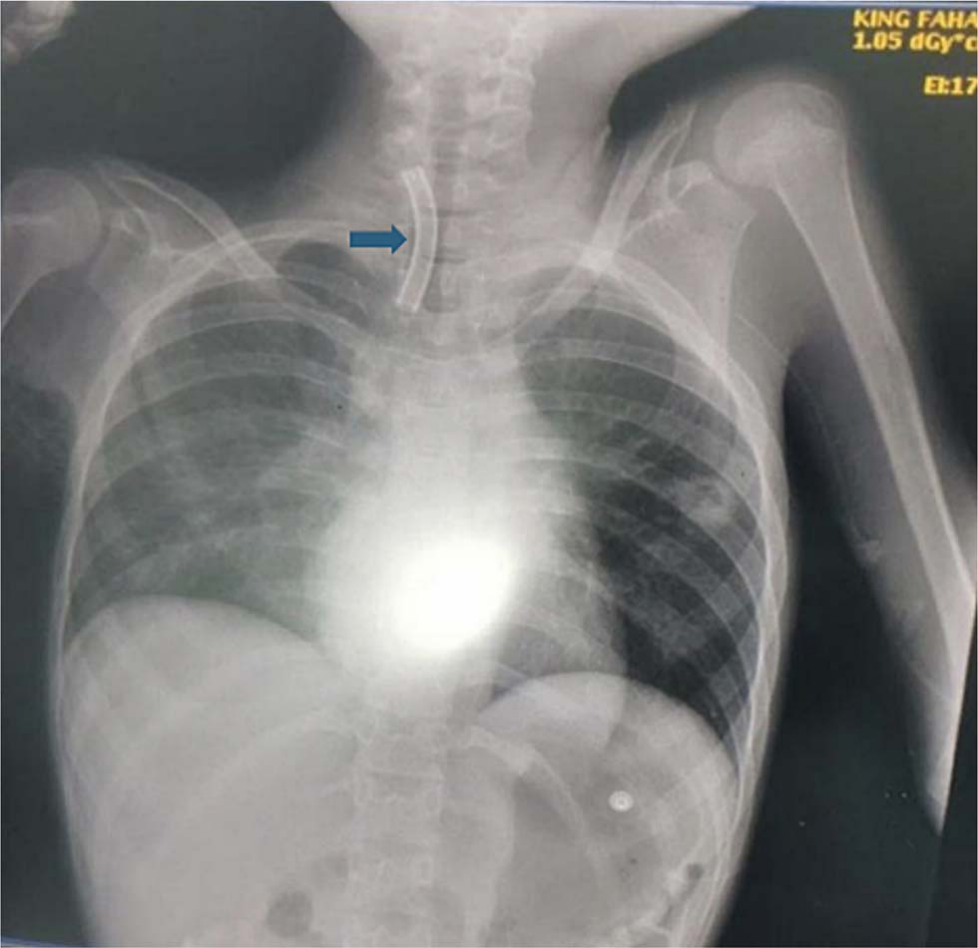
This figure demonstrates the patient chest X-ray showing the radio-opaque cannula in the right main bronchus (arrow).

A 10-year-old female patient was diagnosed with cerebral palsy at birth. As her condition progressed, she developed respiratory muscle weakness and was ventilator dependent with tracheostomy for 5 years. While the patient was in the pediatric intensive care unit (PICU), the medical team observed separation of the tracheostomy wing and cannula dislodgement. Upon arrival for 5 min, the patient showed signs of respiratory distress and oxygen saturation was ~80%. The tracheostomy opening was almost closed, and the oxygen saturation continued to decrease. An immediate chest radiograph was obtained to locate the dislodged cannula. The cannula had migrated into the right main bronchus (Fig. 1).

**Figure 2:**
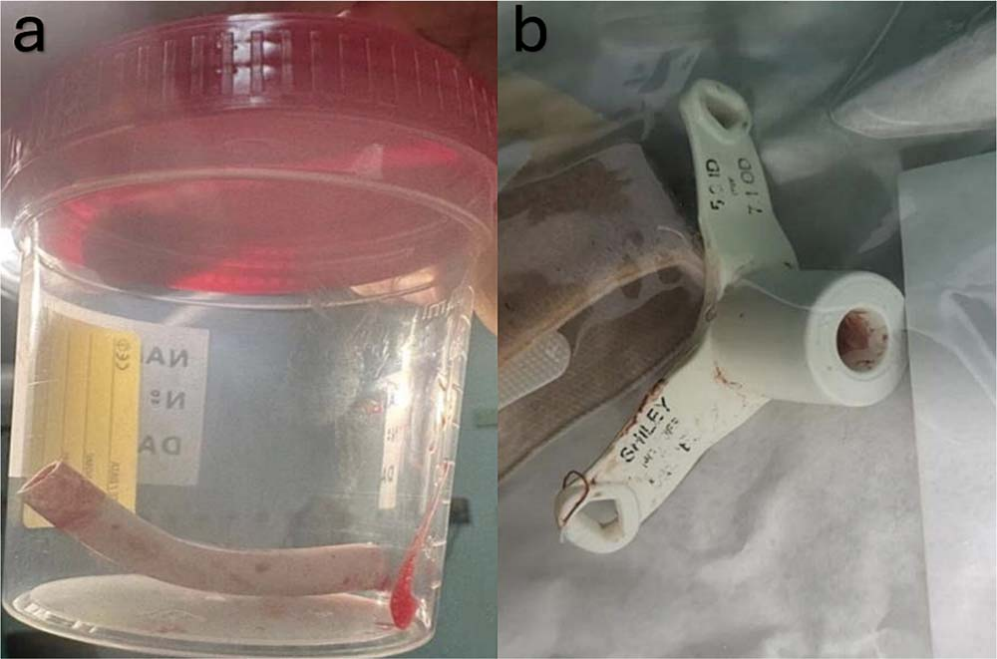
(a) Demonstrates the slipped part of the tracheostomy tube which was extracted from the right main bronchus. (b) Shows the outer and the remaining part of the tracheostomy tube that was broken.

Subsequently, the patient was transferred to the operating room. We monitored the patient closely and immediately secured intravenous access. Spontaneous respiration was initially maintained upon awakening, followed by airway stabilization using a supraglottic airway device. During preparation for bronchoscopy, the patient's oxygen saturation decreased to 70%–65%. We decided to perform. A vertical midline incision at the preexisting stoma was chosen for direct access to the dislodged material, which is crucial for rapid intervention. The cannula was successfully dislodged and removed by using long forceps (Fig. 2). A new size 5 Shiley TT was inserted to reestablish the airway. The patient's oxygen saturation level returned to the baseline level. Thus, hemostasis was achieved. Examination of the foreign body revealed the entire TT length. The patient’s postoperative course was uneventful.

Postoperatively, the patient was closely monitored in the PICU using an oxygen mask over a new TT. Oxygen saturation remained stable at 100%. The patient continued to undergo regular follow-up.

The preceding case underscores how fractured TTs can lead to life-threatening complications if not promptly identified and managed. To contextualize this rare complication within existing knowledge, we now transition to a broader discussion that synthesizes the findings from our case with insights from the relevant literature. This approach aims to highlight both the unique aspects of our case and general trends observed in similar cases.

## Discussion

Having outlined the clinical challenges encountered in this case, we contextualized our experience within the broader spectrum of reported TT fractures to highlight the importance of the early identification and management of such rare complications. Fractured TTs present as foreign bodies in the airway and represent a rare but serious complication that requires immediate attention and intervention [4]. A literature review of 11 relevant papers from PubMed, MEDLINE, and Web of Science over the past 5 years was conducted. By comparing our case study's findings with the synthesized current knowledge, we aimed to highlight both the unique aspects of our case and the common trends observed in the literature (Table 1).

**Table 1:** Time explanation, time from tracheostomy placement till the event of fracture tracheostomy tube.

Author, year	Patient age	Type of tracheostomy tube (material)	Location of fracture	Time	Location of foreign body	Management
Afiadigwe *et al.*, 2024 [4]	56-year-old	Metallic (silver alloy)	Junction between the shaft and flange	6 years	The left main bronchus	Rigid bronchoscopy via stoma
Kashoob *et al.*, 2020 [5]	29-year-old	Metallic double-lumen (Jackson)	Junction between shaft and flange	14 years	The right main bronchus	Rigid bronchoscopy
Mahattanasakul *et al.*, 2022 [6]	Average age 52.75 years	Metallic (silver, stainless steel)	Mid-shaft of outer tube (75%) Junction between tube and neck plate (25%)	Average 24 days	Right tracheobronchus (50%) Right main bronchus (25%) Left main bronchus (25%)	Rigid bronchoscopy via stoma (75%) Flexible bronchoscopy (25%)
Parida *et al.*, 2020 [7]	Mean 10.18 years	Metallic tube (45.5%), PVC (36.4%), Fuller’s tube (18.1%)	Tube–neck plate junction (90.9%)	Average 2 years	Trachea (54.4%) Right main bronchus (36.4%) Carina (9.1%)	Rigid bronchoscopy
Shnaydman *et al.*, 2022 [8]	53- and 74-year-old	Polyvinyl chloride (PVC)	Flange separated from outer cannula	Immediate	N/A (detected prior to migration)	Orotracheal intubation and open tracheostomy
Singhal *et al.*, 2022 [9]	7-year-old	Nonmetallic (PVC)	Junction between neck plate and tube	10 months	Left main bronchus	Rigid bronchoscopy
Tezcan *et al.*, 2023 [10]	61-year-old	PVC	Junction of cannula and neck plate	5 days posttracheostomy	Left main bronchus	Rigid ventilating bronchoscope and forceps through tracheostomy stoma
Chakma *et al.*, 2023 [11]	41-year-old	PVC	Distal part of tube	2 years	Left main bronchus	Flexible fibreoptic bronchoscope under monitored anesthesia care and HFNC
Waindeskar *et al.*, 2022 [12]	35-year-old	PVC	Whole shaft fractured (entire tube dislodged)	11 years	Right main bronchus	Bronchoscopic retrieval, extended tracheostomy incision
Atwood *et al.*, 2022 [13]	19-year-old	Silicone	Junction between neck plate and cannula	6 months	Distal to stoma, proximal to carina	Rigid bronchoscopy under general anesthesia, transoral removal
Jain *et al.*, 2024 [14]	3-year-old	PVC	Junction of stem and neck plate	Unknown	Mid-trachea	Neck exploration under GA using supraglottic airway device

### Etiology of fractured tracheostomy tubes

The causes behind TT fractures and subsequent airway migration are multifaceted. Extended use of a single TT, beyond 3 months in this patient's case posed a significant risk due to material fatigue, a common issue where repeated stress degrades the tube's structural integrity. These complications can occur immediately after tube insertion and require immediate intervention. Documented cases show that the flange of the TT, the part that sits against the neck, can fracture or become dislodged after percutaneous tracheostomy, a less invasive procedure for creating an airway. The outer cannula separates from the flange shortly after insertion, requiring orotracheal intubation and open revision surgical tracheostomy [8]. Additional contributing factors include material degradation associated with aging, repetitive cleaning and sterilization processes (particularly those employing bleaching agents), mechanical stress, and tissue–tube interactions. These elements have been consistently identified in the literature as primary causative factors [5, 6]. Furthermore, stomal complications such as narrowing, infection, and peristomal granulation tissue may contribute to TT fracture [7]. Manufacturing defects, improper handling [4, 6], and patient-specific factors such as forceful coughing, underlying respiratory distress, trauma, and prone positioning have also been implicated [10]. After exploring the multifaceted etiology of TT fractures, including contributing factors, we focused on the varied clinical presentations observed in patients.

These etiological factors, by compromising the structural integrity of TTs, predispose patients to a spectrum of clinical manifestations ranging from asymptomatic to severe respiratory distress.

### Clinical presentation of tube fractures

Following the exploration of the causes of TT fractures, we delve into their clinical manifestations to understand the underlying causes of patient symptoms. Patients with fractured TTs may present with a spectrum of symptoms ranging from being entirely asymptomatic to experiencing severe respiratory distress [4, 8]. Common manifestations include sudden onset of unexplained and persistent cough, dyspnea, choking episodes, stridor, and obvious respiratory distress [6]. Physical examination may reveal reduced breath sounds, cyanosis, tachypnea, tachycardia, fever, and hypoxemia [7, 8, 10].

### Diagnostic approaches for identifying fractures

Chest radiography plays a crucial role in confirming the diagnosis and identifying the precise location of a fractured tube. This serves as an invaluable diagnostic aid for the management of this condition [14].

Before discussing the management strategies for fractured TTs, it is important to summarize the diagnostic approaches that guide treatment decisions. Chest radiography plays an essential role in confirming TT fractures by identifying their location within the airway. Bronchoscopy is often employed for direct visualization of fractures or fragments.

### Management strategies for fractured tubes

With a comprehensive understanding of the clinical presentations and diagnostic approaches for TT fractures, we now focus on critical steps in their management. The cornerstone of managing a fractured TT is expeditious prevention of additional complications. Bronchoscopy is the primary intervention in the management of fractured TTs. Depending on the specific case, a rigid or flexible bronchoscope may be employed, with each chosen on the basis of factors such as the location of the fracture and the patient’s condition. The choice between accessing via the tracheostomy stoma or the oral cavity depends on the position of the tube and the clinician's assessment of the safest approach [5, 9, 11, 14]. Optical forceps are often used to grasp and extract fragments [5]. Neck exploration is necessary in certain scenarios. However, thoracotomy and bronchotomy are reserved for rare and complex cases [4, 12, 14].

Before concluding, it is essential to address the identified gaps in our current understanding of TT fractures, such as the long-term effects of cleaning agents and optimal tube replacement schedule. Therefore, future studies should focus on these aspects. This will not only enhance our comprehension but also significantly contribute to the development of evidence-based guidelines for preventing such complications.

## Conclusion

Based on our case findings and the literature review, it is clear that fractured TTs, although rare, require immediate attention. Future research directions such as the long-term effects of cleaning agents and optimal tube replacement schedules should be discussed. It is crucial to emphasize the importance of early detection and prompt management, as evidenced in our case.

## Data Availability

The data used to support the findings of this study are available upon request.
